# A unified descriptor framework for hydrogen storage capacity and equilibrium pressure in interstitial hydrides

**DOI:** 10.1039/d6sc03089k

**Published:** 2026-05-25

**Authors:** Seong-Hoon Jang, Di Zhang, Xue Jia, Hung Ba Tran, Linda Zhang, Ryuhei Sato, Yusuke Hashimoto, Yusuke Ohashi, Toyoto Sato, Kiyoe Konno, Shin-ichi Orimo, Hao Li

**Affiliations:** a Advanced Institute for Materials Research (WPI-AIMR), Tohoku University Sendai 980-8577 Japan jang.seonghoon.b4@tohoku.ac.jp shin-ichi.orimo.a6@tohoku.ac.jp li.hao.b8@tohoku.ac.jp; b Unprecedented-scale Data Analytics Center, Tohoku University Sendai 980-8578 Japan; c Frontier Research Institute for Interdisciplinary Sciences (FRIS), Tohoku University Sendai 980-8577 Japan; d Department of Materials Engineering, The University of Tokyo Tokyo 113-8656 Japan; e Institute for Materials Research, Tohoku University Sendai 980-8577 Japan; f Institute of Fluid Science, Tohoku University Sendai 980-8577 Japan

## Abstract

Hydrogen is a promising energy carrier, yet its practical deployment is limited by the lack of storage materials that simultaneously achieve high storage capacity (*w*) and practical equilibrium pressure at room temperature (*P*_eq,RT_). Interstitial metal hydrides offer fast kinetics and favorable thermodynamics (high *P*_eq,RT_) but suffer from intrinsically low *w*. Here, we establish a physically interpretable, data-driven framework to uncover descriptor–property relationships in interstitial hydrides using a curated database of pressure-composition-temperature measurements (Digital Hydrogen Platform, *DigHyd*) and white-box symbolic regression. Strikingly, the analysis reveals a clear separation of governing mechanisms, in which *w* is governed by geometric and lattice conditions, captured by the average atomic radius (〈*r*_M_〉) and average thermal conductivity (〈*κ*〉), with an optimal regime of 〈*r*_M_〉 ∼ 1.47* *Å and relatively low 〈*κ*〉. In contrast, *P*_eq,RT_ is governed by elastic properties, captured by the average shear modulus (〈*G*〉) and average Poisson's ratio (〈*ν*〉), reflecting the role of lattice rigidity and mechanical compliance. These relationships are translated into compositional optimization pathways that follow the descriptor trends above, enabling the design of candidate materials with enhanced *w* under practical equilibrium conditions (*P*_eq,RT_ ∼ 0.1 MPa). This work establishes a general, interpretable strategy for physics-informed design of energy materials systems.

## Abstract 2

Hydrogen is a promising energy carrier, yet its practical deployment is limited by the lack of storage materials that simultaneously achieve high storage capacity (*w*) and practical equilibrium pressure at room temperature (*P*_eq,RT_).

## Graphical abstract

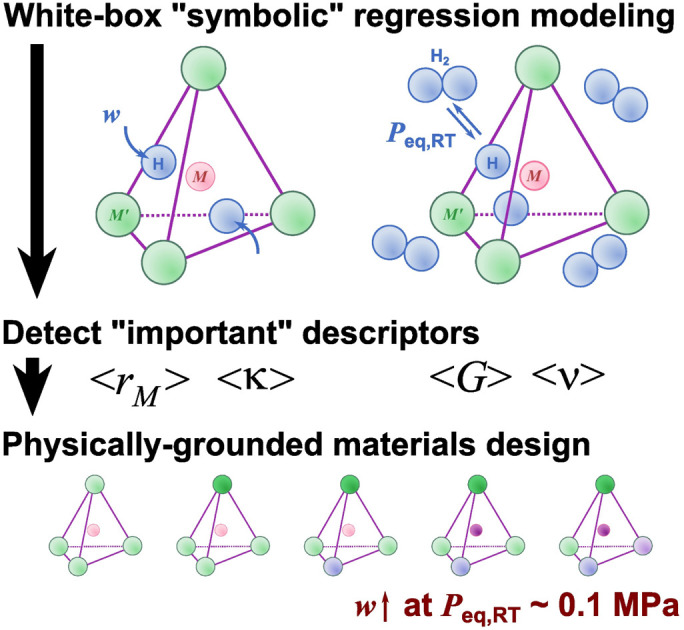

## Introduction

Hydrogen is widely regarded as a key enabler for carbon-neutral energy systems, primarily due to its high energy density by mass and the absence of carbon emissions upon use.^1,2^ Despite these advantages, its widespread application in fuel cells and related technologies remains constrained by the lack of storage solutions that are simultaneously compact, safe, and reversible.^3^ Among the various approaches proposed to address this challenge, solid-state hydrogen storage in metal hydrides has attracted considerable attention because of its high volumetric density, cyclability, and compatibility with engineered systems.^4–6^ Metal hydrides, including representative systems such as MgH_2_, Mg_2_NiH_4_, FeTiH_2_, PdH_0.6_, and LaNi_5_H_6_, span a wide thermodynamic range depending on their bonding characteristics.^7–15^ Saline-type hydrides composed of light elements (*e.g.*, MgH_2_) can achieve high gravimetric capacities but typically require elevated temperatures for hydrogen release,^13,16^ whereas interstitial hydrides based on transition or heavier metals (*e.g.,* LaNi_5_H_6_) exhibit fast kinetics and favorable equilibrium pressures, albeit with intrinsically limited hydrogen storage capacity.^11^

Despite decades of extensive investigation, the compositional landscape of hydride-forming alloys remains far from fully explored. While a vast number of binary and multicomponent systems are theoretically accessible, only a limited fraction has been experimentally synthesized and evaluated. This challenge is further exacerbated by the absence of predictive frameworks that are both quantitatively accurate and physically interpretable, which hinders rational materials design. Although recent machine learning approaches have shown promise in accelerating property prediction,^17–19^ they often rely on relatively small or inconsistently curated datasets and employ black-box models that provide limited insight into the underlying physicochemical mechanisms. To overcome these limitations, we previously developed the Digital Hydrogen Platform (*DigHyd*: https://www.dighyd.org), a curated database constructed through large-scale extraction of experimental pressure-composition-temperature (PCT) data from the literature.^20,21^ Building on this dataset, symbolic regression was performed using a white-box modeling approach, enabling the construction of explicit relationships between materials descriptors and key hydrogen storage metrics, namely gravimetric capacity (*w*) and equilibrium pressure at room temperature (*P*_eq,RT_).^22,23^ This approach identified a compact set of physically meaningful descriptors, including contributions from atomic mass, electronic structure, and packing characteristics, which govern hydrogen storage behavior. In particular, systems containing light elements were found to favor higher hydrogen capacity, whereas electronic and structural descriptors play a dominant role in determining equilibrium pressure. Within this descriptor space, beryllium (Be)-containing alloys emerged as promising candidates; however, their practical applicability is severely limited by toxicity and associated handling constraints.^24^

**Fig. 1:**
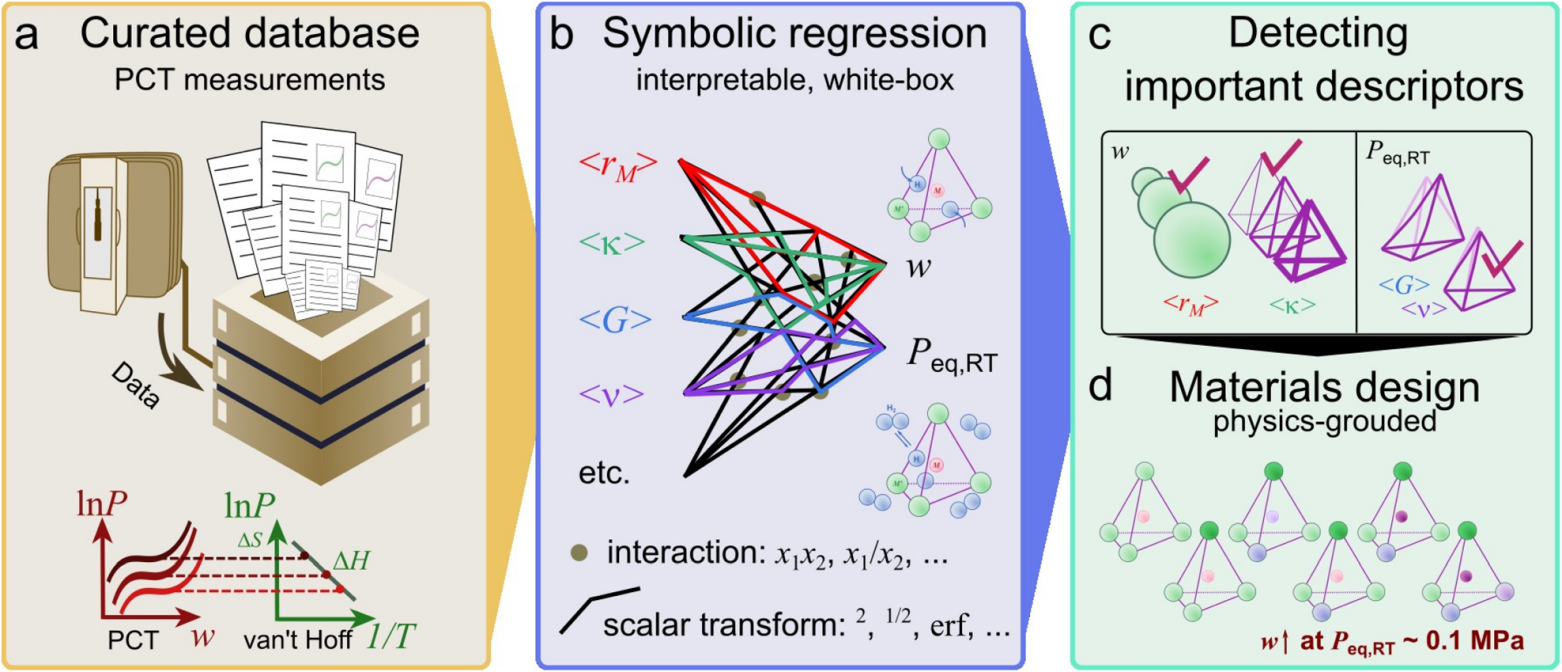
Workflow of the present study for physics-informed materials design of interstitial hydrides. (a) Construction of a curated database of pressure-composition-temperature (PCT) measurements (*DigHyd*). (b) Development of interpretable, white-box symbolic regression models linking materials descriptors to hydrogen storage properties, including gravimetric capacity (*w*) and equilibrium pressure at room temperature (*P*_eq,RT_). (c) Identification of key descriptors governing *w* and *P*_eq,RT_ within the interstitial hydride space. (d) Translation of descriptor–property relationships into materials design guidelines, enabling the optimization of *w* under practical equilibrium pressure conditions (*P*_eq,RT_ ∼ 0.1 MPa).

These considerations motivate a shift toward more practical materials systems, as schematically illustrated in Fig. 1. Fig. 1a shows the construction of a curated database of PCT measurements, *DigHyd*, which serves as the foundation for this study. Building on this dataset, Fig. 1b presents the development of interpretable, white-box symbolic regression models that link materials descriptors to key hydrogen storage properties, *w* and *P*_eq,RT_. Focusing on interstitial hydrides, we aim to identify the key descriptors governing both *w* and *P*_eq,RT_ by extracting physically meaningful descriptors from the symbolic models (see Fig. 1c). Finally, as shown in Fig. 1d, these descriptor–property relationships are translated into materials design guidelines that enable enhanced *w* under practical operating conditions. In particular, we target equilibrium pressures near ambient conditions, *P*_eq,RT_ ∼ 0.1 MPa, corresponding in this study to the window of −1.5 log_10_[MPa] < log_10_*P*_eq,RT_ < −0.5 log_10_[MPa].

## Results

### Data curation and feature construction for interstitial hydrides

Often, PCT experiments involve multi-phase materials, which can introduce unintended noise in regression modeling. To mitigate this issue, we filtered the dataset and retained only single-phase or near-single-phase cases. Here, “near-single-phase” refers to materials in which a single phase accounts for more than 80 wt% of the total. As a result, the total number of entries is 706. However, not all entries include multi-temperature measurements required to determine *P*_eq,RT_. Consequently, the number of data points (*n*_data_) used for modeling *w* and *P*_eq,RT_ are 706 and 299, respectively. This reduction primarily reflects the limited availability of consistent multi-temperature PCT measurements in the literature, despite the broader abundance of hydrogen capacity data. Within this context, the present framework is designed to extract robust and physically meaningful insights from limited but high-quality datasets. For BCC-type alloys exhibiting double plateaus, the plateau corresponding to practical operating conditions near ambient pressure was consistently selected, as the lower plateau is often located far below atmospheric pressure and is not practically accessible.

**Fig. 2:**
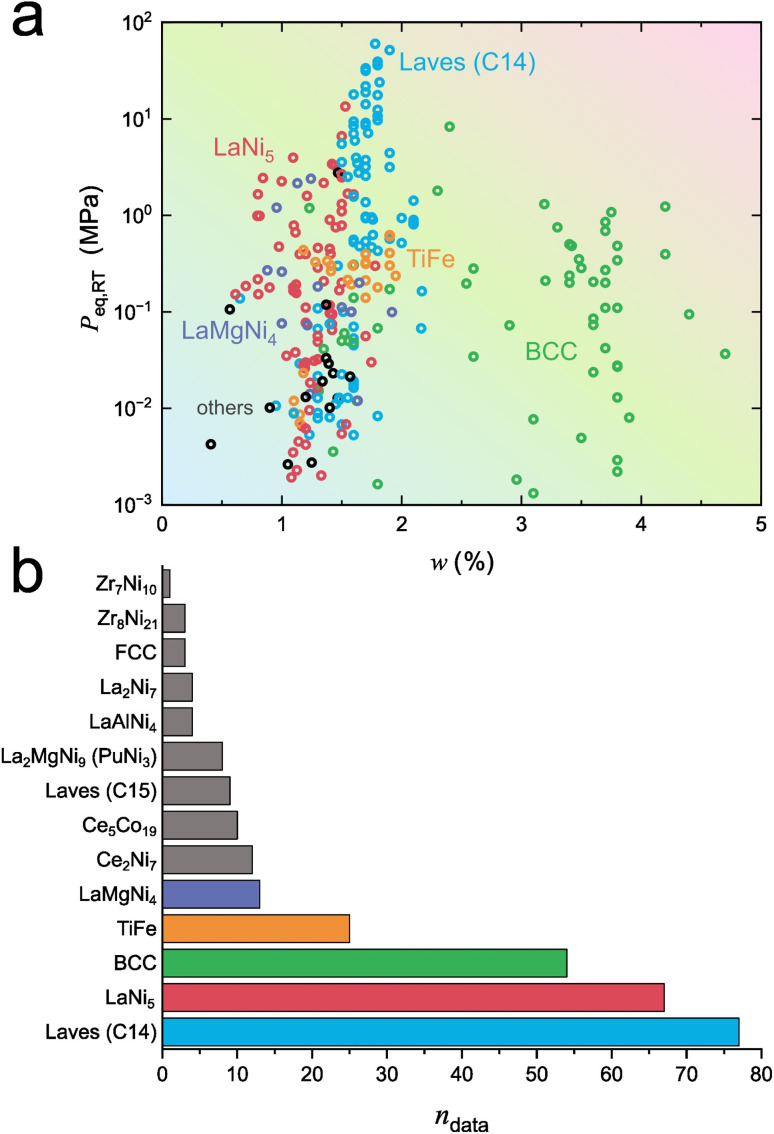
Data distribution and feature construction for interstitial hydrides. (a) Distribution of data points in the *w* − *P*_eq,RT_ map for entries with multi-temperature PCT data. (b) Number of data points (*n*_data_) for each structural class.

Fig. 2a shows the distribution of data points in the two-dimensional *w* − *P*_eq,RT_ materials map for cases where multi-temperature PCT data are available. Most structural classes with different parent structures, such as Laves (C14), LaNi_5_, LaMgNi_4_, and TiFe, are located in the low-*w* region (*w* < 2.5%), whereas the BCC class extends into the higher-capacity region (2 < *w* < 5%). However, BCC metals and alloys generally exhibit two distinct plateaus in their PCT curves, with the lower plateau typically located far below atmospheric pressure at room temperature, thereby limiting the practical accessibility of their full *w*.^25^Fig. 2b presents *n*_data_ for each reported class. Notably, three classes, Laves (C14), LaNi_5_, and BCC, out of 14 classes account for *n*_data_ = 198, corresponding to 66.2% of the dataset with multi-temperature PCT measurements (*n*_data_ = 299), which is close to the Pareto principle.

For symbolic regression modeling, features were extracted as candidate descriptors for *w* and *P*_eq,RT_. The full list of features and their denotations is provided in Table 1; hereafter, the denotations are omitted for simplicity. A total of 57 features were constructed, comprising 18 chemical, physical, and structural features applied to their averaged values ⋯, standard deviations *σ*(⋯), and skewness *r*(⋯), along with three additional compositional features (*R*_Tr_, *R*_Tr(IV)_, and *R*_Tr+RE_). For example, for a compound *A*_*a*_*B*_*b*_*C*_*c*_, the averaged atomic mass is defined as *M* = (*aM*_*a*_ + *bM*_*b*_ + *cM*_*c*_)/(*a* + *b* + *c*), where *M*_*x*_ denotes the atomic mass of element *x*. Structural descriptors such as Ω, Ω_*σ*_, and *V*_p_ require the definition of coordination polyhedra *XY*_*n*_ (*X*, *Y* = *A*, *B*, and *C* crystallographic sites; *X*-centered). The maximum cutoff distance for metal–metal pairs was set to 3.5 Å, corresponding to the shoulder of the first peak in the radial distribution function averaged across crystal structures (see the section “Averaged Radial Distribution Function of Metal-alloy Parent Structures and the Construction of *XY*_*n*_ Polyhedra” in the SI). In addition, the heatmap of Pearson correlation coefficients (*r*_col_) among all feature pairs is provided in the section “Pearson Correlation Heatmap” in the SI.

**Table 1:** Chemical, physical, structural, and compositional properties of constituent elements of interstitial metal hydrides for regression models, given as features for symbolic regression modeling

Properties	Description	Unit
*χ*–*χ*_H_	Difference in electronegativity between metal atom and hydrogen	—
*M*	Atomic mass	g mol^−1^
*n*_ve_	The number of valence electrons. For s/p-block and d/f-block, electrons in the shells of *n*_p_*s* and *n*_p_*p*, and in those of *n*_p_*s* and (*n*_p_ − 1)*d* are counted, respectively; *n*_p_ is the highest principal quantum number	—
*r*_M_	Metallic radius	Å
*ρ*	Density	g cm^−3^
*ρ*_mol_	Molar density, defined as *ρ*/*M*	mol cm^−3^
*η*_fM_	Metallic filling rate (per unit volume), defined as 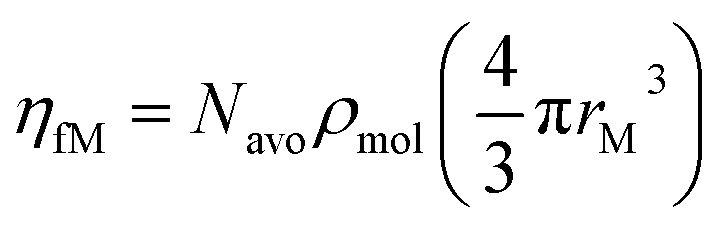 where *N*_avo_ is the Avogadro's constant	—
*B*	Bulk modulus	GPa
*G*	Shear modulus	GPa
*ν*	Poisson's ratio	—
*κ*	Thermal conductivity	W m^−1^ K^−1^
*α*	Thermal expansion coefficient (linear not volumetric)	K^−1^
*θ*_D_	Debye temperature	K
*χ*_m_	Molar susceptibility	m^3^ mol^−1^
*n*_c_	Coordination number to neighbor metal atoms within 3.5 *Å* for each crystallographic site (*A*, *B*, and *C*). For example, in the compound LaMgNi_4_, *A*, *B*, and *C* correspond to La, Mg, and Ni, respectively	—
Ω	Solid angle per face constructed in polyhedra of *XY*_*n*_ (*X*, *Y* = *A*, *B*, and *C* crystallographic sites; *X*-centered; and *X*–*Y* distance shorter than 3.5 *Å*). Estimated from the parent structures listed in Fig. 2b	Radian
Ω_*σ*_	Standard deviation across Ω for each crystallographic site (*A*, *B*, and *C*). Estimated from the parent structures listed in Fig. 2b	Radian
*V*_p_	Volume of polyhedra of *XY*_*n*_ for each crystallographic site (*A*, *B*, and *C*). Estimated from the parent structures listed in Fig. 2b	Å^3^
⋯	Average over the constituent metal ions	Same with the unit of ⋯
*σ*(⋯)	Standard deviation over the constituent metal ions	Same with the unit of ⋯
*r*(⋯)	Skewness over the constituent metal ions	—
*R*_Tr_	Compositional fraction of transition metal elements	—
*R*_Tr(IV)_	Compositional fraction of the fourth-row transition metal elements (Sc, …, Zn)	—
*R*_Tr+RE_	Compositional fraction of transition and rare-earth metal elements (La, …, Lu)	—

### Key descriptors governing *w* and *P*_eq,RT_

**Fig. 3:**
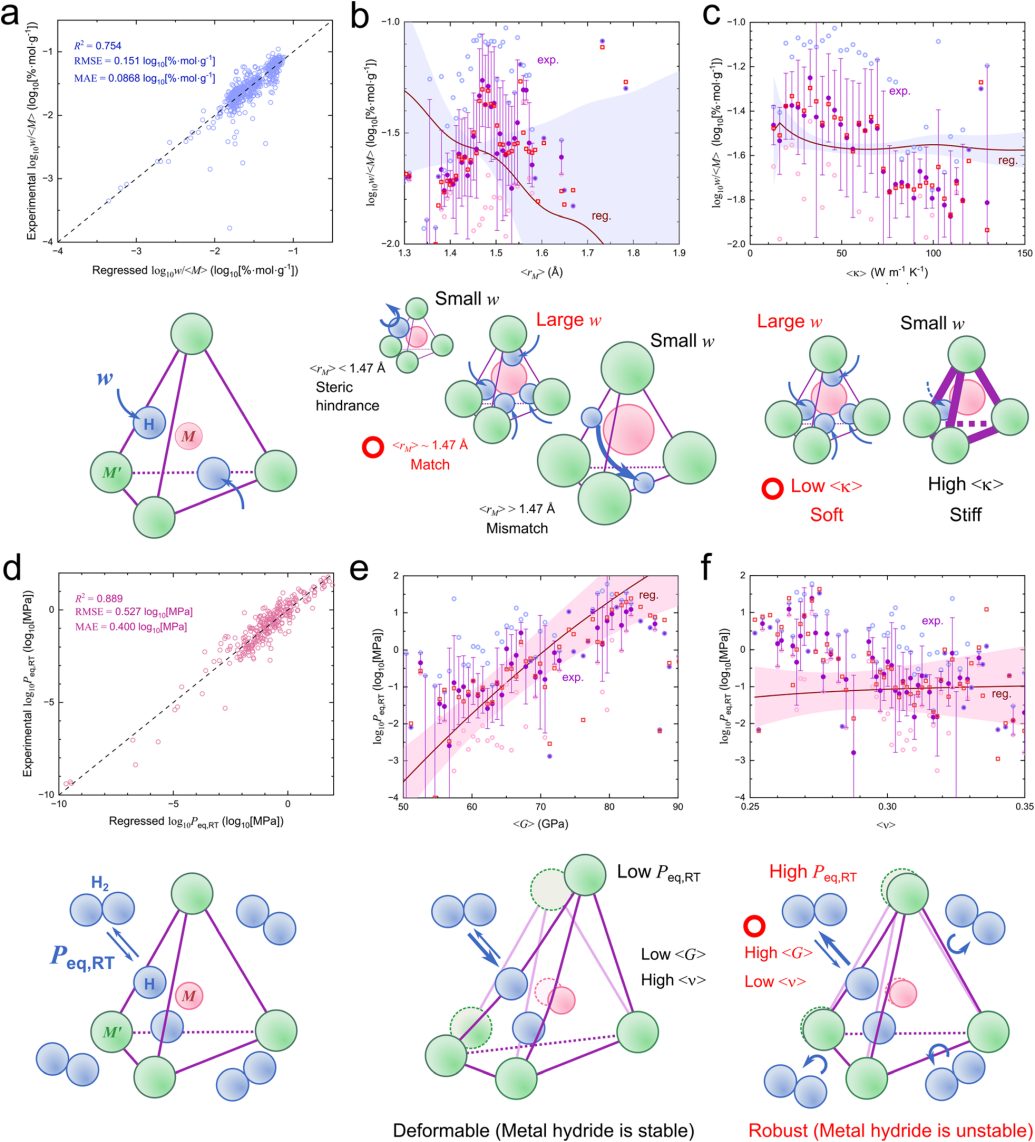
Predictive performance and descriptor analysis for hydrogen capacity and equilibrium pressure. (a) Parity plot for log_10_(*w*/*M*) using the symbolic regression model. (b) and (c) Partial dependence plots (PDPs) for the two most important descriptors governing log_10_(*w*/*M*). (d) Parity plot for log_10_*P*_eq,RT_. (e) and (f) PDPs for key descriptors governing log_10_*P*_eq,RT_. In the PDPs of (b), (c), (e), and (f), the regression curve (“reg.”) is obtained by fixing all other features to their average values. Experimental data (“exp.”) are partitioned into 100 bins along the horizontal axis, where markers denote the minimum (represented by a magenta circle), average (a purple circle; with standard deviation represented by a purple bar), and maximum values (a cyan circle) within each bin, as well as the averaged regressed value (a red rectangule). The shaded region represents the variation across individual symbolic models within the ensemble. Schematic illustrations are presented below each panel to define *w* and *P*_eq,RT_ and to illustrate the relationships between the descriptors and the corresponding properties. As materials design principles, the ideal conditions for achieving high *w* and high *P*_eq,RT_ are highlighted in red and indicated by red circles.

Given the strong predictive performance of the white-box modeling approach we adopted (see the section “Benchmarking of Regression Models” in the SI),^19^ further symbolic regression models were reconstructed for the target metrics log_10_(*w*/*M*) and log_10_*P*_eq,RT_, using the full dataset without reserving a separate test set. Fig. 3a demonstrates the strong predictive performance of the model for log_10_(*w*/*M*), achieving *R*^2^ = 0.754, RMSE = 0.151 log_10_[% mol g^−1^], and MAE = 0.0868 log_10_[% mol g^−1^] over the entire dataset (*n*_data_ = 706). Fig. 3b and c present the partial dependence plots (PDPs) for two most important descriptors for *w*. In each case, the PDP curve is obtained by fixing all other features to their average values. As a result, the experimental and regressed data points, which reflect the full compositional effects and multicollinearity among features (see the section “Pearson Correlation Heatmap” in the SI), do not necessarily follow the PDP curve. Importantly, PDPs isolate the effect of each descriptor by fixing all others, thereby revealing intrinsic relationships beyond correlations in the dataset. Nevertheless, comparing the PDP trends with the corresponding data points provides insight into the underlying physics governing the target metrics.

In Fig. 3b, the PDP alone suggests that smaller 〈*r*_M_〉, which is often associated with lower atomic mass, favors higher *w*. However, in real materials, excessively small 〈*r*_M_〉 can instead induce steric constraints, requiring lattice expansion to accommodate interstitial hydrogens. As a result, an optimal value emerges around 〈*r*_M_〉 ∼ 1.47 Å, whereas larger values lead to expanded interstitial sites that are unable to effectively stabilize hydrogen, likely due to geometric mismatch between the interstitial cage size and the effective size required for hydrogen accommodation. Notably, in the BCC structure, 〈*r*_M_〉 = 1.47 Å yields a geometric maximum hard-sphere radius of 0.43 Å for interstitial hydrogen in tetrahedral sites. In Fig. 3c, the PDP suggests that lower thermal conductivity 〈*κ*〉, which is linked to electronic structure near the Fermi level and the strength of metallic bonding, and typically associated with softer lattice structures, correlates with higher *w*. Here, 〈*κ*〉 is interpreted not as an isolated causal factor, but as an effective descriptor reflecting coupled electronic-structure and lattice-related characteristics of the host metal framework. In particular, softer lattice environments can more readily accommodate lattice expansion upon hydrogen insertion, thereby facilitating higher hydrogen uptake. In practice, however, *κ* exhibits a more nuanced influence. In contrast, for 〈*κ*〉 > 110 W m^−1^ K^−1^, *w* shows little variation, indicating that the effect of *κ* becomes saturated in this regime. Taken together, these results indicate that *w* is maximized when 〈*r*_M_〉 is tuned to approximately 1.47 Å in conjunction with relatively low 〈*κ*〉.

Fig. 3d demonstrates the strong predictive performance of the model for log_10_*P*_eq,RT_, achieving *R*^2^ = 0.889, RMSE = 0.527 log_10_[MPa], and MAE = 0.400 log_10_[MPa] over the entire dataset (*n*_data_ = 299). Fig. 3e and f present the PDPs for two most important descriptors for log_10_*P*_eq,RT_. In Fig. 3e, both the PDP and experimental and regressed data points show good agreement. A higher 〈*G*〉 indicates a more rigid lattice, which increases the elastic energy penalty associated with interstitial hydrogen insertion and thereby destabilizes hydride formation, leading to higher *P*_eq,RT_. In Fig. 3f, the PDP suggests that the influence of 〈*ν*〉 on *P*_eq,RT_ is relatively weak. In real materials, however, *P*_eq,RT_ decreases rapidly when *ν* is below approximately 0.3 and becomes nearly constant above this threshold. This behavior suggests that materials with higher 〈*ν*〉 are more mechanically compliant and can more readily accommodate lattice deformation during hydride formation, consistent with the negative correlation between 〈*G*〉 and 〈*v*〉 shown in the section “Pearson Correlation Heatmap” in the SI. Taken together, these results indicate that lattice rigidity plays a central role in governing equilibrium pressure, with stiffer crystal structures leading to higher *P*_eq,RT_. To further assess the robustness of the model across different structural classes, a class-resolved sensitivity analysis was performed (see the section “Class-resolved Model Performance and Sensitivity Analysis” in the SI), confirming that the predictive performance is not dominated by any single class.

### Materials design for high *w* at practical *P*_eq,RT_

**Fig. 4:**
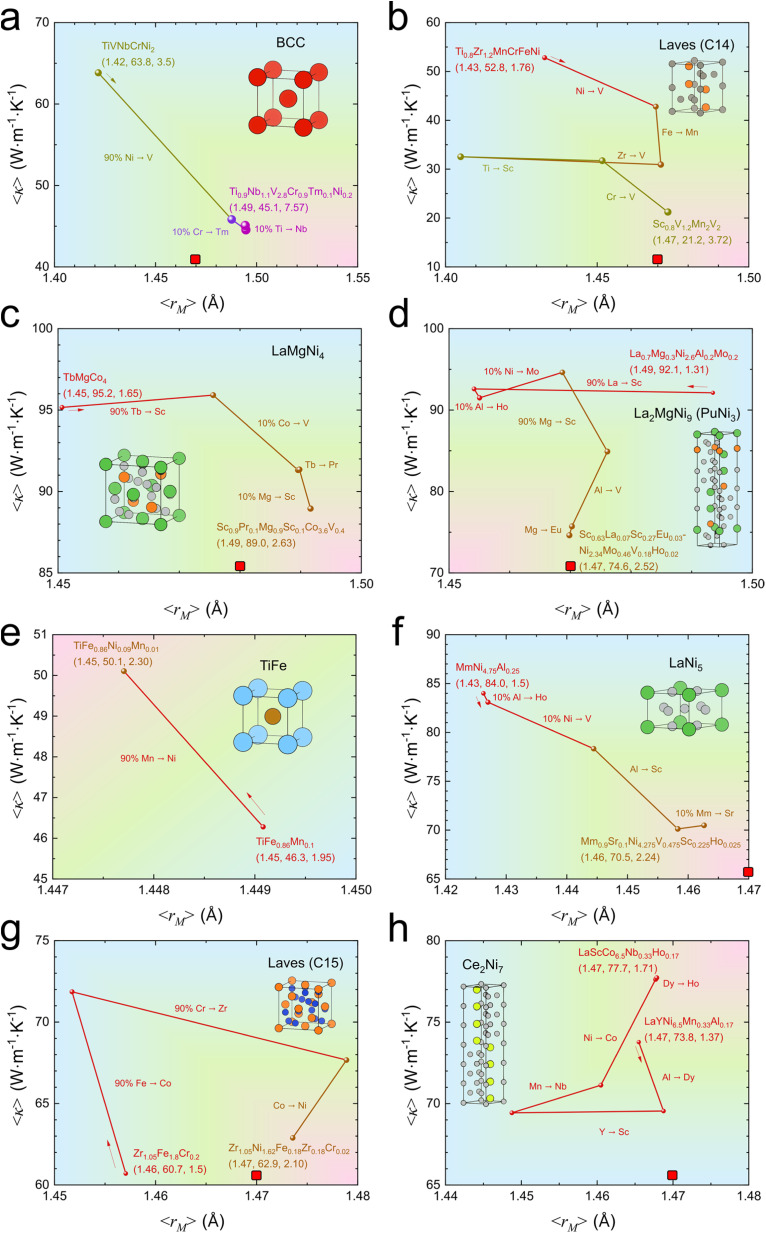
Materials design pathways toward high hydrogen capacity under practical equilibrium pressure, mapped onto the 〈*κ*〉-〈*r*_M_〉 plot. Optimization trajectories for representative parent structure classes: (a) BCC,^26^ (b) Laves (C14),^27^ (c) LaMgNi_4_,^28^ (d) La_2_MgNi_9_ (PuNi_3_),^29^ (e) TiFe,^30^ (f) LaNi_5_,^31^ (g) Laves (C15),^32^ and (h) Ce_2_Ni_7_.^33^ Each panel shows the compositional optimization pathway guided by the symbolic models within the target window −1.5 log_10_[MPa] < log_10_*P*_eq,RT_ < −0.5 log_10_[MPa]. The marker size and color, as well as the line color at each step, are scaled by *w*. Initial and optimized compositions are represented by descriptor-performance vectors *p* = (〈*r*_M_〉/Å, 〈*κ*〉/(W m^−1^ K^−1^), *w*/%), together with intermediate substitution steps. The red rectangule in each panel indicates a shared design target identified by symbolic models, where 〈*r*_M_〉 converges to approximately 1.47 Å with reduced 〈*κ*〉. In most cases, 〈*κ*〉 either decreases or remains nearly constant, consistent with adherence to the design rule of 〈*r*_M_〉 → ∼1.47 Å. The parent crystal structures are also visually represented. The color gradient is used as a visual guide to indicate the overall direction of materials design trends, rather than representing a quantitative colormap. The reliability of the optimized candidate compositions was further evaluated using ensemble uncertainty metrics (see the section “Uncertainty Assessment of Optimized Candidate Materials” in the SI), indicating that the proposed candidates lie within a low-uncertainty prediction regime. These compositions should be interpreted as physically guided design candidates rather than directly synthesizable materials, and further experimental validation will be required.

All *DigHyd* entries with multi-temperature PCT data (*n*_data_ = 299) were subjected to optimization toward higher *w* under the target condition of *P*_eq,RT_ ∼ 0.1 MPa, guided by the symbolic models. For each parent structure class, the composition with the highest predicted *w* within the window −1.5 log_10_[MPa] < log_10_*P*_eq,RT_ < −0.5 log_10_[MPa] was selected, resulting in 8 representative cases. Fig. 4a–h are arranged in descending order of the optimized *w*, corresponding to the following structure types: BCC > Laves (C14) > LaMgNi_4_ > La_2_MgNi_9_ (PuNi_3_) > TiFe > LaNi_5_ > Laves (C15) > Ce_2_Ni_7_.

As an illustrative example, Fig. 4a shows the optimization of the BCC-type alloy TiVNbCrNi_2_, initially characterized by a descriptor-performance vector *p* = (〈*r*_M_〉/Å, 〈*κ*〉/(W m^−1^ K^−1^), *w*/%) = (1.42, 63.8, 3.5).^26^ The optimization proceeds through a multi-step compositional pathway: first, 90% of Ni is replaced with V to yield TiVNbCrV_1.8_Ni_0.2_; next, 10% of Cr is substituted with Tm, resulting in TiVNbCr_0.9_Tm_0.1_V_1.8_Ni_0.2_; finally, 10% of Ti is replaced with Nb, leading to the composition Ti_0.9_Nb_0.1_VNbCr_0.9_Tm_0.1_V_1.8_Ni_0.2_. This sequence yields the optimized composition Ti_0.9_Nb_1.1_V_2.8_Cr_0.9_Tm_0.1_Ni_0.2_, with final *p* = (1.49, 45.1, 7.57). Similar symbolic-model-guided optimization systematically shifts compositions toward enhanced *w* across other structure types^27–33^ by modulating 〈*r*_M_〉 and 〈*κ*〉 (Fig. 4b–h). Here, all descriptors are updated self-consistently with composition and are not fixed to their average values, in contrast to the PDP analysis in Fig. 3.

Across all optimized cases, two consistent design principles emerge in most cases, represented by red rectangules in Fig. 4a–h. First, 〈*r*_M_〉 converges toward an optimal value of approximately 1.47 Å, represented by dashed magenta lines. Second, 〈*κ*〉 either decreases or remains nearly unchanged during optimization. These trends directly reflect the descriptor–property relationships identified in Fig. 3b and c, demonstrating that the symbolic models capture transferable design rules for achieving high *w* under practical *P*_eq,RT_ conditions. Although the predicted values of *w* themselves may be subject to uncertainty due to their extrapolative nature, the proposed optimization pathways provide physically grounded and clear design directions, based on the white-box symbolic models. Also, this design pathway can be further enhanced through integration with a closed-loop discovery framework,^34,35^ in which symbolic models are iteratively refined using synthesis and measurement data guided by the optimization trajectories, thereby enabling more accurate, real-world-relevant predictions.

## Discussion

The present study reveals that hydrogen storage behavior in interstitial hydrides can be described by a small number of physically interpretable descriptors that govern distinct aspects of performance. In particular, *w* is governed by the interplay between geometric and electronic-lattice factors, captured by 〈*r*_M_〉 and 〈*κ*〉. The existence of an optimal 〈*r*_M_〉 ∼ 1.47 Å reflects a geometric constraint on hydrogen accommodation, while lower 〈*κ*〉, associated with softer lattices, facilitates hydrogen incorporation. These results indicate that maximizing *w* requires simultaneous optimization of both interstitial geometry and lattice adaptability. Atomic size effects have long been recognized as an important factor in hydrogen storage alloys, particularly through their influence on interstitial site size and lattice geometry; it was shown that variations in atomic radius modulate the available void space for hydrogen accommodation, thereby affecting hydrogen storage capacity *w*.^36^ In this context, the emergence of 〈*r*_M_〉 as a key descriptor for *w* is consistent with established physical understanding. In contrast, the identification of 〈*κ*〉 for *w* is to the best of our knowledge, less explored in the literature.

In contrast, *P*_eq,RT_ is primarily governed by elastic properties, described by 〈*G*〉 and 〈*ν*〉. A higher 〈*G*〉 increases the elastic penalty for hydrogen insertion, leading to higher equilibrium pressure, whereas larger *ν* reflects greater mechanical compliance and lowers *P*_eq,RT_. Thus, *P*_eq,RT_ is fundamentally controlled by the elastic response of the host lattice. These descriptors provide complementary representations of lattice rigidity and deformability rather than independent contributions. This also can be understood in the context of the van't Hoff equation, 
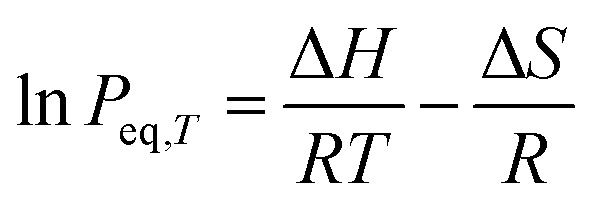
, where *P*_eq,*T*_ is the equilibrium pressure at temperature *T*, and Δ*H* and Δ*S* are the enthalpy and entropy changes associated with hydride formation. For many metal-hydrogen systems, Δ*S* is largely dominated by the loss of gas-phase entropy when molecular hydrogen is incorporated as atomic hydrogen into the lattice. Consequently, variations in *P*_eq,RT_ are primarily governed by changes in Δ*H*. In this context, 〈*G*〉 and 〈*ν*〉 reflecting the elastic energy penalty is consistent with a prior study demonstrating that lattice strain has been shown to modify the effective Δ*H* and shift *P*_eq,*T*_ over several orders of magnitude by altering the elastic energy associated with hydrogen insertion.^37^ Furthermore, 〈*G*〉 has recently been reported as a key descriptor governing Δ*H* in the formation of BCC high-entropy alloy monohydrides, based on a machine learning study.^38^

Thus, we emphasize that these relationships are consistent mainly with established physical understanding, and the present analysis provides a quantitative framework to support and organize these insights. Here, it should be noted that the present descriptor framework is most applicable to systems undergoing isostructural hydrogenation. In materials with non-isostructural phase transitions, additional thermodynamic and structural factors may become dominant.

Taken together, these results establish a clear separation of roles between descriptors governing capacity and thermodynamics. While *w* is controlled by geometric-electronic factors, *P*_eq,RT_ is dictated by elastic constraints. This separation provides a physically transparent and actionable framework for materials design under practical operating conditions. Importantly, these descriptor relationships are consistently observed across different structure types, indicating that they define a transferable design space. Compositions across multiple classes converge toward 〈*r*_M_〉 ∼ 1.47 Å with reduced 〈*κ*〉, demonstrating the robustness of the identified design principles. The present framework is expected to be most reliable within the probed compositional and structural domains of interstitial hydrides. Application beyond this space may require incorporation of additional descriptors to capture different bonding regimes, phase transformations, or thermodynamic contributions not present in interstitial hydrides.

These findings should also be understood in the broader context of descriptor discovery across hydrogen storage materials. In our previous study, we showed that across a global materials space, including both interstitial and saline hydrides, *w* and *P*_eq,RT_ are strongly governed by elemental electronegativity, and can therefore impose an intrinsic trade-off between capacity and equilibrium pressure.^19^ This indicates that, at the global scale, electronegativity acts as a unifying descriptor controlling hydrogen storage behavior across fundamentally different bonding types. In contrast, the present work focuses on the local materials space of interstitial hydrides, where more specific descriptors emerge, reflecting finer structural and mechanical effects. Thus, while global electronegativity-driven trends may introduce trade-offs, the local descriptor framework identified here provides a route to navigate and potentially mitigate these trade-offs for simultaneous optimization of high *w* and practical *P*_eq,RT_. Together, these results highlight a “hierarchical” descriptor framework, in which global trends are governed by simple chemical parameters, while local optimization requires more detailed, physically specific descriptors.

More broadly, the present results demonstrate that symbolic regression can resolve coupled physical mechanisms into interpretable descriptor relationships, enabling a transition from empirical exploration toward mechanism-informed materials design. This approach provides a generalizable strategy for descriptor-based materials design in hydrogen-related systems. Furthermore, such a framework is expected to be directly applicable to saline hydrides and, more broadly, to systems beyond hydrogen storage, including hydride-based solid-state electrolytes for batteries.^39^

## Conclusion

In this work, we established a physically interpretable, data-driven framework to uncover descriptor–property relationships governing hydrogen storage behavior in interstitial hydrides using curated PCT data and symbolic regression. A small set of physically meaningful descriptors was identified, revealing that *w* and *P*_eq,RT_ are governed by fundamentally distinct mechanisms. In particular, *w* is controlled by geometric and electronic-lattice factors, whereas *P*_eq,RT_ is dictated by elastic properties of the host lattice. These insights were further translated into actionable materials design guidelines, enabling the identification of compositions with enhanced hydrogen capacity under practical operating conditions. The resulting design principles are transferable across different structure types and compositional spaces, demonstrating the robustness of the descriptor-based framework. Beyond hydrogen storage, the present approach provides a general strategy for integrating interpretable machine learning with physically grounded materials design, with potential applications extending to other energy materials.

## Methods

### *DigHyd* database

The Digital Hydrogen Platform (*DigHyd*) is a rigorously curated database of hydrogen storage materials constructed through an AI-assisted, human-in-the-loop literature mining framework. As described in ref. 21, *DigHyd* integrates experimentally reported data from more than 4000 literature sources, comprising over 30 000 curated data entries on hydrogen storage properties and thermodynamic parameters. Importantly, the database spans a wide range of material classes beyond conventional metal hydrides, including interstitial hydrides, complex hydrides, ionic (saline) hydrides, multi-component and destabilized systems, as well as porous materials such as metal–organic frameworks. This breadth enables systematic analysis across fundamentally different hydrogen storage mechanisms. The curated data include gravimetric hydrogen capacity (*w*), as well as the enthalpy (Δ*H*) and entropy (Δ*S*) changes associated with hydrogenation reactions, primarily defined as 
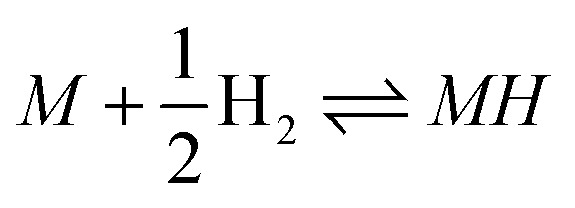
.

### Symbolic modeling

While the algorithmic details of the white-box symbolic regression modeling package (*GoodRegressor*) are described in ref. 23, a brief summary of its use in this study is provided here. For each symbolic model constructed using the *Regressor* module described in ref. 20, the dataset was randomly split into training and validation sets with a ratio of 8 : 2. Given the set of candidate descriptors listed in Table 1, the number of possible model combinations including interaction terms is given as 1.95 × 10^463^ for the 109 default scalar transforms provided in the *Regressor* module.

Model construction was therefore performed in a staged manner. Starting from models with 20 active independent variables, the number of active variables was iteratively reduced one by one (20 → 19 → 18 → ⋯ → 1), while progressively allowing more complex interaction terms (*i.e.*, increasing interaction depth). At each stage, candidate models were evaluated, and the model with the highest *R*^2^ over the entire dataset was selected.

All models were generated under the so-called *full Fisher condition*, whereby the *p*-values of both the overall model (*F*-test) and all individual coefficients (*t*-tests) were required to be less than 0.05. For each target metric (log_10_(*w*/*M*) and log_10_*P*_eq,RT_), 10 symbolic models were constructed and subsequently combined into a single “stacking-ensembled” model. Key descriptors were then identified by examining features that consistently appeared across the 10 symbolic models, as well as by evaluating the magnitude of their contributions through *z*-scored coefficients. In particular, descriptors associated with terms exhibiting large average *z*-scored coefficient magnitudes across the ensemble were considered to play dominant roles in determining the target properties.

To ensure model reliability, the standard deviation across the regressed values in the ensemble was constrained to be below 0.5 log_10_[% mol g^−1^] and 0.7 log_10_[MPa] for log_10_(*w*/*M*) and log_10_*P*_eq,RT_, respectively. Predictions exceeding these thresholds were discarded. In the 5-fold benchmark tests, the discard ratios were approximately 1% and 10% for log_10_(*w*/*M*) and log_10_*P*_eq,RT_, respectively. Notably, when models were constructed using the entire dataset, no data points were discarded, as all predictions satisfied the ensemble consistency criteria. Representative examples of symbolic model ensembles, their error distributions, and comparisons across different target metrics (log_10_*w* and log_10_(*wM*)) are provided in the section “Symbolic Models: Formulation and Error Analysis” in the SI.

### Materials design

For materials optimization, the *Designer* module described in ref. 23 was employed, using the *DigHyd* dataset as input. During the search for optimization pathways from existing experimental compositions, only candidate compositions yielding ensemble standard deviations below 0.5 log_10_[% mol g^−1^] and 0.7 log_10_[MPa] for log_10_(*w*/*M*) and log_10_*P*_eq,RT_, respectively, were considered. For the final optimized compositions, a stricter criterion was applied: only those with ensemble standard deviations below 0.1 log_10_[% mol g^−1^] were retained for log_10_(*w*/*M*).

Compositional modifications were performed through controlled substitution operations while preserving the original stoichiometry. Specifically, full substitution, 90% substitution, and 10% substitution of constituent metal elements were allowed. Candidate substitution elements were required to satisfy two criteria relative to the original element: the differences in electronegativity and metallic radius must not exceed 0.5 and 0.5 Å, respectively.

### Crystal structure visualization

Crystal structures were visualized using VESTA.^40^

## Code availability

The source code supporting materials prediction and design in this study is openly available at https://github.com/JerryGarcia1995/OxygenIonConductor. The repository contains the general symbolic regression framework used in this study.

## Author contributions

H. Li and S.-i. Orimo conceived and supervised the project. S.-H. Jang carried out conceptualization, data curation, formal analysis, methodology development, software implementation, validation, and visualization, and wrote the original draft of the manuscript. D. Zhang contributed to data curation, investigation, methodology, and software development. X. Jia, H. B. Tran, and K. Konno contributed to data curation and investigation. L. Zhang, R. Sato, and Y. Hashimoto reviewed and edited the manuscript. Y. Ohashi and T. Sato contributed to conceptualization and manuscript review and editing. All authors discussed the results and approved the final manuscript.

## Conflicts of interest

There are no conflicts to declare.

## Supplementary Material

SC-OLF-D6SC03089K-s001

## Data Availability

The data can be accessed in the Digital Hydrogen Platform (*DigHyd*: https://www.dighyd.org). Supplementary information (SI): Radial distribution function averaged over metal-alloy parent structures for hydrogen storage and the construction of *XY*_*n*_ polyhedra, Pearson correlation heatmap, benchmarking of regression models, class-resolved model performance and sensitivity analysis, uncertainty assessment of optimized candidate materials, and symbolic models: formulation and error analysis. See DOI: https://doi.org/10.1039/d6sc03089k.
