# Supplementary material for: A unified descriptor framework for hydrogen storage capacity and equilibrium pressure in interstitial hydrides
Source: Chem Sci. 2026 May 25;17(27):13396–406. doi: 10.1039/d6sc03089k (PMC13225154; doi:10.1039/d6sc03089k)
Supplement: SC-017-D6SC03089K-s001 [file SC-017-D6SC03089K-s001.pdf]

Supplementary Information (SI) for Chemical Science.
This journal is © The Royal Society of Chemistry 2026

Supplementary Information

A unified descriptor framework for hydrogen storage capacity and equilibrium pressure in interstitial hydrides

Seong-Hoon Jang[*1, 2], *Di Zhang*[1,3], *Xue Jia*[1], *Hung Ba Tran*[1], *Linda Zhang*[1,3], *Ryuhei Sato*[4], *Yusuke Hashimoto*[3], *Yusuke Ohashi*[5], *Toyoto Sato*[5], *Kiyoe Konno*[6], *Shin-ichi Orimo*[*1, 5], *and Hao Li*[*1]

[1] Advanced Institute for Materials Research (WPI-AIMR), Tohoku University, Sendai 980-8577, Japan

[2] Unprecedented-scale Data Analytics Center, Tohoku University, Sendai 980-8578, Japan

[3] Frontier Research Institute for Interdisciplinary Sciences (FRIS), Tohoku University, Sendai 980-8577, Japan

[4] Department of Materials Engineering, The University of Tokyo, Tokyo 113-8656, Japan

[5] Institute for Materials Research, Tohoku University, Sendai, 980-8577

[6] Institute of Fluid Science, Tohoku University, Sendai, 980-8577, Japan

* Corresponding authors:

jang.seonghoon.b4@tohoku.ac.jp (S.-H. Jang)

shin-ichi.orimo.a6@tohoku.ac.jp (S. Orimo)

li.hao.b8@tohoku.ac.jp (H. Li)

Contents:

- Radial Distribution Function Averaged over Metal-alloy Parent Structures for Hydrogen Storage and the Construction of XY_n Polyhedra

- Pearson Correlation Heatmap

- Benchmarking of Regression Models

Details of Conventional Machine Learning Approaches

- Class-resolved Model Performance and Sensitivity Analysis

- Uncertainty Assessment of Optimized Candidate Materials

- Symbolic Models: Formulation and Error Analysis

Symbolic Model for $\log_{10}(w/\langle M\rangle)$

Symbolic Model for $\log_{10} P_{eq,RT}$

Stacking-ensembled Symbolic Models for $\log_{10} w$ and $\log_{10}(w\langle M\rangle)$

Error Distributions of Stacking-ensembled Models for $\log_{10}(w/\langle M\rangle)$ and $\log_{10} P_{eq,RT}$

Radial Distribution Function Averaged over Metal-alloy Parent Structures for Hydrogen Storage and the Construction of XY_n Polyhedra

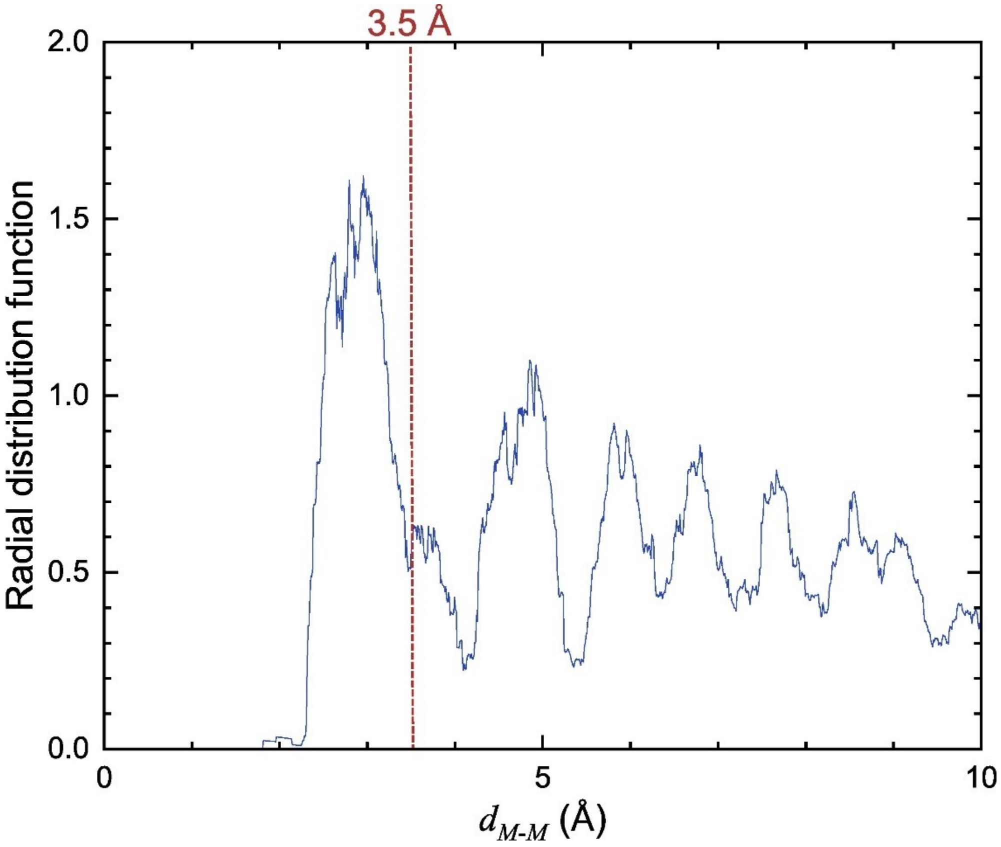

Supplementary Figure 1. Averaged radial distribution functions of metal-alloy parent structures. Radial distribution function averaged over representative metal-alloy parent structures used in this study. The vertical red line at 3.5 Å indicates the cutoff distance adopted for defining neighboring metal atoms in the construction of coordination polyhedra.

In **Supplementary Figure 1,** The first major peak corresponds to nearest-neighbor metallic bonding, while the shoulder region around 3.5 Å represents the upper bound of local coordination environments across different structures. Based on this observation, a cutoff distance of 3.5 Å was selected to consistently define neighboring atoms for structural descriptor construction. This choice ensures that the coordination polyhedra capture the relevant local environments while avoiding inclusion of more distant, weakly interacting atoms.

For the construction of polyhedra XY_n (where X and Y are metal atoms, with X at the center), all Y atoms within a distance of 3.5 Å from X were selected. Three-dimensional convex hulls were then generated. To achieve this, all possible triangular faces $\left(\binom{n}{3}\right)$ were enumerated and subsequently evaluated to determine whether they belonged to the outermost surface or the interior of the polyhedron, based on the orientation of their perpendicular vectors. Subsequently, the included angles between all adjacent faces of the convex hull were examined. If two adjacent faces were found to be coplanar (i.e., lying in the same plane), they were merged into a single unified face. This integration process was iteratively repeated until no further coplanar faces remained. This procedure ensures that the polyhedra are uniquely defined in all cases. Following construction, the validity of each polyhedron was confirmed by verifying that the sum of the solid angles of all faces equals 4π.

Pearson Correlation Heatmap

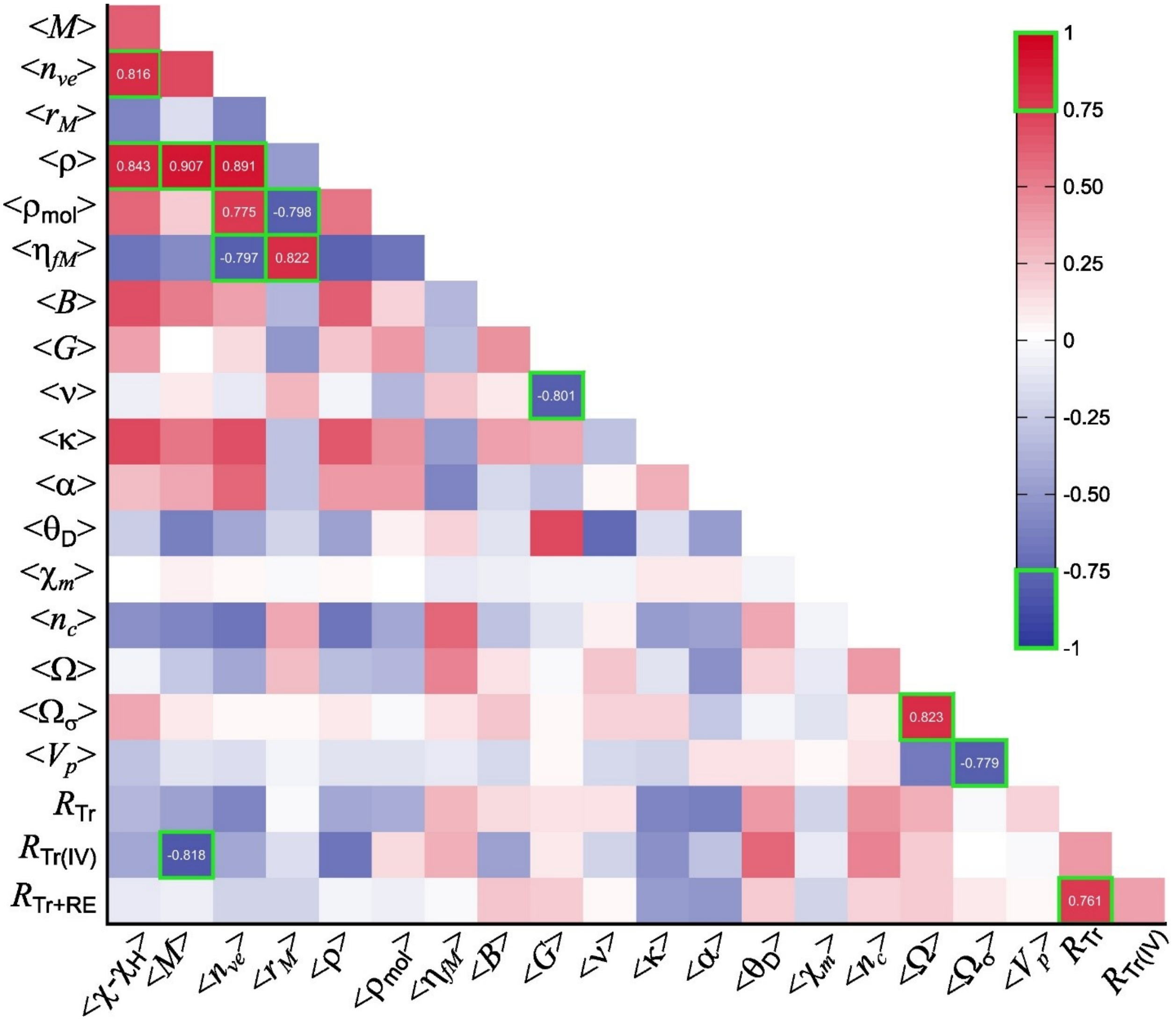

Supplementary Figure 2 Heatmap of Pearson correlation coefficients (r_{col}) among the selected descriptors. Strong correlations ($|r_{col}| > 0.75$) are highlighted.

Supplementary Figure 2 shows the heatmap of Pearson correlation coefficients (r_{col}) among all feature pairs. Several pairs exhibit strong correlations ($|r_{col}| > 0.75$), indicating intrinsic coupling among descriptors. For example, the physical descriptors $\langle G \rangle$ and $\langle \nu \rangle$ show a strong negative correlation ($r_{col} = -0.801$), reflecting the fundamental mechanical response of solids in which

materials with higher G tend to resist transverse deformation and therefore exhibit lower ν. The interdependence patterns provide a physical basis for the descriptor analysis presented in the main text.

Benchmarking of Regression Models

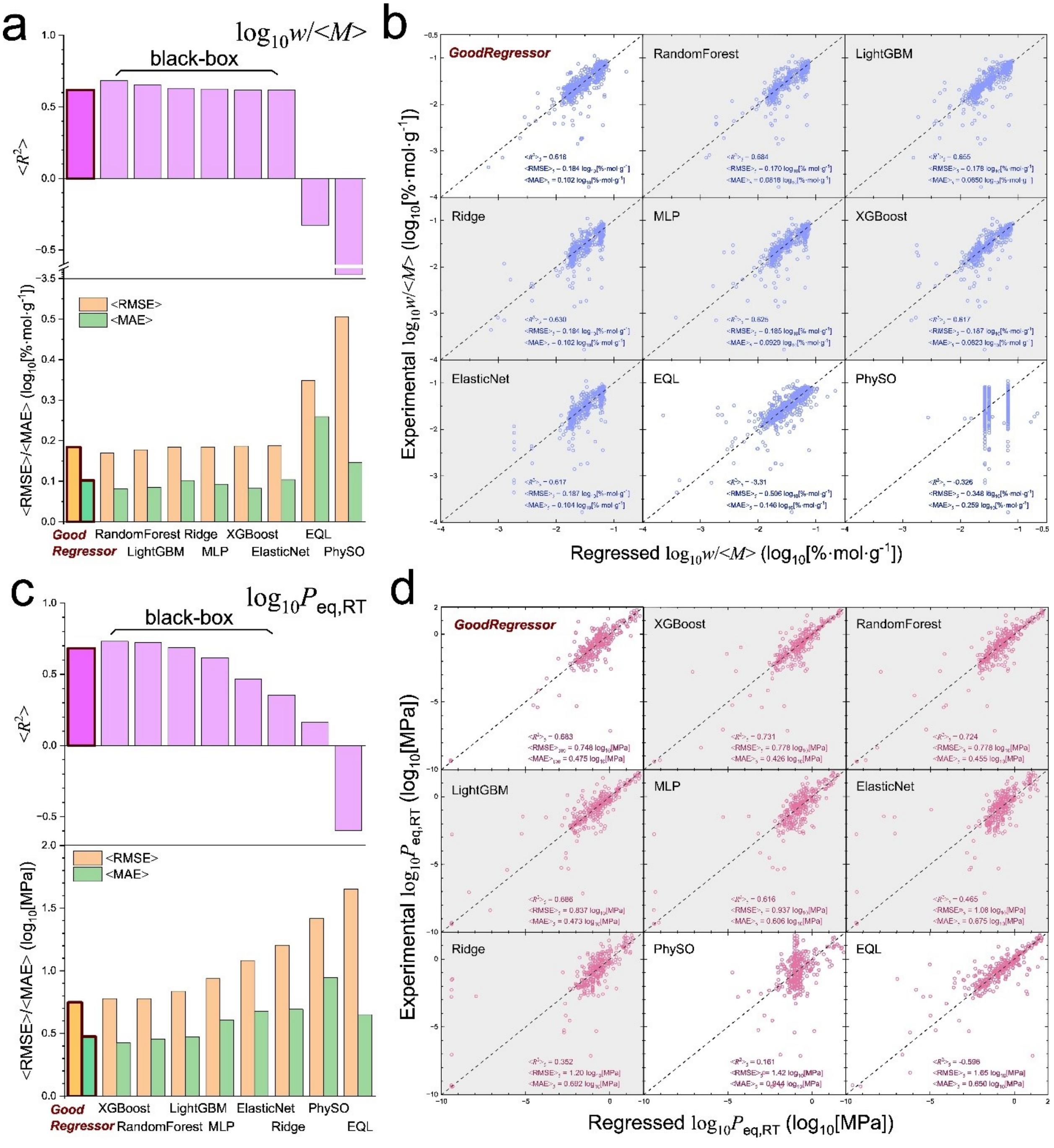

Supplementary Figure 3 Benchmark performance of *GoodRegressor* and other machine learning models. Comparison of stacking-ensembled symbolic regression models generated by using *GoodRegressor*[S1] with conventional machine learning approaches, Ridge,[S2] ElasticNet,[S3] MLP,[S4] RandomForest,[S5] XGBoost,[S6] LightGBM,[S7] EQL,[S8] and F-SO,[S9] for predicting (a, b) w and (c, d)

$P_{eq,RT}$. (a) and (c) show averaged benchmark metrics across five different test folds, that is, coefficient of determination ($\langle R^2 \rangle_5$), root mean square error ($\langle \mathrm{RMSE} \rangle_5$), and mean absolute error ($\langle \mathrm{MAE} \rangle_5$), and present schematic illustrations defining w and $P_{eq,RT}$. (b) and (d) present parity plots comparing experimental and predicted values for each model. In (b) and (d), the black-box models are illustrated as gray panels for visual distinction.

To assess whether *GoodRegressor*, a white-box symbolic regression framework,[S1] can simultaneously achieve high predictive accuracy and interpretability, benchmark tests were performed for two target metrics: $\log_{10}(w/\langle M \rangle)$ and $\log_{10} P_{eq,RT}$. The comparison included black-box models, Ridge,[S2] ElasticNet,[S3] MLP,[S4] RandomForest,[S5] XGBoost,[S6] and LightGBM,[S7] as well as other white-box baselines, EQL[S8] and Phy-SO,[S9] as illustrated in **Supplementary Figure 3**. Technical details are provided in subsequent sections. All models were evaluated using 5-fold cross-validation with hyperparameter optimization, employing identical data splits to ensure fair comparison. For instance, in the *GoodRegressor* case, a fold (20%) was held out as the test set in each iteration, while the remaining data were used for training and validation. Within this subset, 10 independent random $8{:}2$ train-validation splits were generated to construct a stacking-ensembled symbolic regression model. Repeating this procedure across all folds yielded 50 individual symbolic models and 5 ensemble models in total, corresponding to overall data usage of $64\ \%$ for training, $16\ \%$ for validation, and $20\ \%$ for testing.

In **Supplementary Figure 3a**, the benchmark results for the $\log_{10}(w/\langle M \rangle)$ dataset are presented in terms of three metrics: the averaged coefficient of determination ($\langle R^2 \rangle_5$), the averaged root mean square error ($\langle \mathrm{RMSE} \rangle_5$), and the averaged mean absolute error ($\langle \mathrm{MAE} \rangle_5$) across the five validation folds. **The *GoodRegressor* model achieved performance comparable to state-of-the-art black-box machine learning models while outperforming all other white-box baselines.** Specifically, it achieved $\langle R^2 \rangle_5 = 0.618$, $\langle \mathrm{RMSE} \rangle_5 = 0.184 \log_{10}[\% \cdot mol \cdot g^{-1}]$, and $\langle \mathrm{MAE} \rangle_5 = 0.102 \log_{10}[\% \cdot mol \cdot g^{-1}]$. Meanwhile, the other models exhibited performance ($\langle R^2 \rangle_5 \leq 0.684$, $\langle \mathrm{RMSE} \rangle_5 \geq 0.170 \log_{10}[\% \cdot mol \cdot g^{-1}]$, and $\langle \mathrm{MAE} \rangle_5 \geq 0.0818 \log_{10}[\% \cdot mol \cdot g^{-1}]$). The performance ranking among these was as follows: RandomForest > LightGBM > Ridge > MLP

> *GoodRegressor* > XGBoost > ElasticNet > EQL > PhySO. The parity plots in **Supplementary Figure 3b** as well further confirm that the *GoodRegressor* model provides excellent predictive accuracy.

Supplementary Figure 3c presents the benchmark results for the dataset of $\log_{10} P_{eq,RT}$. **Again, the *GoodRegressor* model demonstrated performance comparable to black-box machine learning methods while outperforming other white-box baselines.** Specifically, it achieved $\langle R^2 \rangle_5 = 0.683$, $\langle \mathrm{RMSE} \rangle_5 = 0.748 \log_{10} [MPa]$, and $\langle \mathrm{MAE} \rangle_5 = 0.575 \log_{10} [MPa]$. The other models recorded comparable or lower performance ($\langle R^2 \rangle_5 \leq 0.731$, $\langle \mathrm{RMSE} \rangle_5 \geq 0.778 \log_{10} [MPa]$, and $\langle \mathrm{MAE} \rangle_5 \geq 0.426 \log_{10} [MPa]$). The performance ranking among these was as follows: XGBoost > RandomForest > LightGBM > *GoodRegressor* > MLP > ElasticNet > Ridge > PhySO > EQL. The parity plots in **Supplementary Figure 3d** as well further confirm that *GoodRegressor* provides excellent predictive accuracy. Taken together, *GoodRegressor* represents a unique approach that simultaneously achieves reliable predictive performance and inherent white-box interpretability.

Details of conventional machine learning approaches. To evaluate the predictive performance of various regression algorithms on the dataset, a standardized benchmarking pipeline was implemented in Python. This framework provides a uniform and unbiased comparison between conventional machine-learning and symbolic regression approaches. The evaluation follows a nested cross-validation design with systematic hyperparameter optimization, ensuring fair and reproducible comparison across models. Each model underwent 5-fold nested cross-validation. The details of parameters are given below.

a. Ridge

Core library: scikit-learn

Search parameters: $a \in [10^{-4}, 10^{3}]$

Iterations: 80

b. ElasticNet

Core library: scikit-learn

Search parameters: $a \in [10^{-4}, 10^{1}]$, l_1 ratio $\in [0, 1]$

Iterations: 80

c. MLP

Core library: scikit-learn

Search parameters: Hidden layers: (256, 128), (256, 128, 64), (512, 256, 128); activation: ReLU or tanh; $a \in [10^{-6}, 10^{-2}]$; learning rate $\in [3 \times 10^{-4}, 3 \times 10^{-2}]$; max_iter = 4000

Iterations: 80

d. RandomForest

Core library: scikit-learn

Search parameters: n_estimators ∈ [800, 2000], max_depth ∈ [6, 28], min_samples_split ∈ [2, 12], min_samples_leaf ∈ [1, 6], max_features ∈ [0.3, 0.7]

Iterations: 100

e. XGBoost

Core library: xgboost

Search parameters: n_estimators ∈ [1200, 3000], learning rate ∈ [0.01, 0.2], max_depth ∈ [3, 12], subsample ∈ [0.6, 1.0], reg_lambda ∈ [1, 80], gamma ∈ [10^{-9}, 10^{-1}]

Iteration: 140

f. LightGBM

Core library: lightgbm

Search parameters: n_estimators ∈ [1500, 4000], learning rate ∈ [0.01, 0.2], num_leaves ∈ [31, 255], min_child_samples ∈ [5, 120], feature/bagging fraction ∈ [0.6, 1.0], l_1, l_2 ∈ [10^{-3},10]

Iteration: 140

g. EQL

Epochs: 600

Learning rate: 1×10^{-3}

L_1 penalty: 1×10^{-3} on output weights and projection layers

Activation functions: {sin, cos, exp, log, erf, square, cube, linear, multiplicative interactions}

Term constraint: maximum 20 active symbolic terms via adaptive top-K masking

i. F-SO

Each symbolic search ran for 60 epochs, operating on symbolic operators {mul, add, sub, div, n^2, sqrt, neg, exp, log, sin, cos}.

Class-resolved Model Performance and Sensitivity Analysis

Supplementary Table 1 Class-resolved sensitivity analysis for $\log_{10}(w/\langle M\rangle)$ and $\log_{10} P_{eq,RT}$, including within-class and leave-one-class-out evaluations. Metrics reported are R^2, $RMSE$, MSE, n_{data}, and mean ensemble standard deviation.

Case	n_{data}	R^2	RMSE	MSE	Mean ensemble standard deviation
$\log_{10}(w/\langle M\rangle)$					
all	706	0.820	0.129	0.017	0.044
within Laves C14	141	0.236	0.121	0.015	0.039
within LaNi5	125	0.403	0.108	0.012	0.038
within BCC	220	0.770	0.138	0.019	0.051
within TiFe	35	-0.026	0.132	0.017	0.041
without Laves C14	565	0.844	0.132	0.017	0.046
without $LaNi_5$	581	0.818	0.134	0.018	0.046
without BCC	486	0.706	0.125	0.016	0.041
without TiFe	671	0.827	0.129	0.017	0.045
$\log_{10} P_{eq,RT}$					
all	299	0.901	0.497	0.247	0.308
within Laves C14	80	0.910	0.418	0.175	0.258
within LaNi5	68	0.690	0.490	0.240	0.304
within BCC	57	0.867	0.569	0.324	0.411
within TiFe	26	0.989	0.332	0.110	0.294
without Laves C14	219	0.883	0.523	0.274	0.326
without $LaNi_5$	231	0.917	0.499	0.249	0.309
without BCC	242	0.907	0.479	0.229	0.284
without TiFe	273	0.850	0.510	0.260	0.309

To evaluate the robustness and transferability of the developed models across different structural families, a class-resolved sensitivity analysis was performed for both $\log_{10}(w/\langle M\rangle)$ and $\log_{10} P_{eq,RT}$. The analysis includes (i) within-class performance, where model accuracy is evaluated using only data belonging to a specific structural class, and (ii) leave-one-class-out analysis, where all data from a given class are excluded and the model performance is re-evaluated on the remaining dataset. The structural classes considered include Laves C14, $LaNi_5$, BCC, and TiFe systems.

Model performance is quantified using the coefficient of determination (R^2), root-mean-square error (RMSE), mean squared error (MSE), and the number of data points (n_{data}) in each subset. In addition, the mean ensemble standard deviation is reported as an estimate of prediction uncertainty.

For the **within-class analysis**, the predictive performance varies across structural classes. In particular, classes with limited data or a narrow range of target values may exhibit artificially low R^2 values due to the reduced variance of the reference data, whereas **RMSE and MSE remain largely unaffected.** In contrast, the **leave-one-class-out analysis** shows that the overall model performance remains relatively stable upon exclusion of individual structural classes. **This indicates that the learned relationships are not dominated by any single class, but instead capture generalizable trends across diverse interstitial hydride systems.** Moderate variations in performance are observed for certain classes, reflecting their relative representation and contribution to the overall dataset. **The ensemble standard deviation remains consistent across different subsets, without significant increases in any particular class or exclusion case.** This suggests that the model uncertainty is well-controlled and does not indicate systematic extrapolation behavior for specific structural families. **Overall, this analysis supports the robustness of the proposed descriptor-based models and their applicability across multiple classes of interstitial hydrides.**

Uncertainty Assessment of Optimized Candidate Materials

Supplementary Table 2 Optimized candidate compositions identified for each structural class, along with predicted w, $P_{eq,RT}$, and their corresponding logarithmic values. The ensemble standard deviation is reported as an estimate of prediction uncertainty for each target. The consistently low ensemble standard deviations indicate that the proposed candidates lie within a reliable prediction regime and do not correspond to extreme extrapolations of the model.

Class	Final goal	w (%)	$\log_{10} w$ ($\log_{10}$[%])	ensemble standard deviation ($\log_{10}$[%])	$P_{eq,RT}$ (MPa)	$\log_{10} P_{eq,RT}$ ($\log_{10}[MPa]$)	ensemble standard deviation ($\log_{10}[MPa]$)
BCC	$Ti_{0.9}Nb_{1.1}V_{2.8}Cr_{0.9}Tm_{0.1}Ni_{0.2}$	7.57	0.879	0.0847	0.055	-1.26	0.420
Laves (C14)	$Sc_{0.8}V_{1.2}Mn_2V_2$	3.72	0.571	0.0794	0.193	-0.71	0.645
$LaMgNi_4$	$Sc_{0.9}Pr_{0.1}Mg_{0.9}Sc_{0.1}Co_{3.6}V_{0.4}$	2.63	0.420	0.0768	0.300	-0.52	0.530
La_2MgNi_9 ($PuNi_3$)	$Sc_{0.63}La_{0.07}Sc_{0.27}Eu_{0.03}Ni_{2.34}Mo_{0.46}V_{0.18}Ho_{0.02}$	2.52	0.401	0.0988	0.161	-0.79	0.688
TiFe	$TiFe_{0.86}Ni_{0.09}Mn_{0.01}$	2.30	0.362	0.0610	0.216	-0.67	0.258
$LaNi_5$	$Mm_{0.9}$Sr0.1$Ni_{4.275}V_{0.475}Sc_{0.225}Ho_{0.025}$	2.24	0.351	0.0816	0.073	-1.14	0.230
Laves (C15)	$Zr_{1.05}Ni_{1.62}Fe_{0.18}Zr_{0.18}Cr_{0.02}$	2.10	0.321	0.0861	0.174	-0.76	0.481
Ce_2Ni_7	$LaScCo_{6.5}Nb_{0.33}Ho_{0.17}$	1.93	0.285	0.0932	0.258	-0.59	0.634

To assess the reliability of the optimized candidate materials proposed in **Figure 4** in the main text, the ensemble standard deviations were summarized for both $\log_{10} w$ and $\log_{10} P_{eq,RT}$ in **Supplementary Table 2**. The ensemble standard deviation provides an intrinsic measure of prediction uncertainty, where larger values typically indicate extrapolation beyond the domain of the training data. As shown, the ensemble standard deviations for $\log_{10} w$ remain consistently low across all optimized candidates (typically below 0.10), indicating that w are obtained within a well-supported region of the descriptor space. Similarly, the ensemble standard deviations for $\log_{10} P_{eq,RT}$ remain moderate and comparable to those observed within the training dataset, without exhibiting anomalously large values. **These results suggest that the proposed optimized compositions do not correspond to extreme extrapolations, but rather represent controlled exploration within a reliable prediction regime defined by the model.** Therefore, while the

candidates are obtained through optimization, their predicted properties are supported by the internal consistency of the ensemble model and the imposed uncertainty constraints.

Symbolic Models: Formulation and Error Analysis

In this section, as representative examples, one of the ten symbolic regression models for each target metric ($\log_{10}(w/\langle M \rangle)$ and $\log_{10} P_{eq,RT}$) is provided, and error distributions for both target properties are provided as well. Additionally, “inferior” stacking-ensembled symbolic models for $\log_{10} w$ and $\log_{10}(w\langle M \rangle)$ are represented.

a. Symbolic Model for $\log_{10}(w/\langle M\rangle)$

Supplementary Table 3 An example of symbolic model for $\log_{10}(w/\langle M\rangle)$. Here, $\chi_{\Delta H}=\chi-\chi_H$.

Term index i	Coefficient c_i	Z-scored coefficient $z(c_i)$	Term t_i
1	8.65×10^{-1}	0.498	$\langle\Omega\rangle R_{\mathrm{Tr}}$
2	-1.17	-0.409	$\cos\left(\frac{\pi}{200}\frac{\langle V_p\rangle}{R_{\mathrm{Tr+RE}}}\right)$
3	-1.92×10^{-1}	-0.225	$10^{\frac{\langle B\rangle}{\langle\theta_{\mathrm{D}}\rangle}}$
4	-8.93	-0.221	$\sin\left(\frac{\pi}{10}\langle\chi_{\Delta H}\rangle\langle r_M\rangle\right)\left(\mathrm{erf}\left(\langle\nu\rangle R_{\mathrm{Tr(IV)}}-\frac{1}{20}\right)\right)^{-\frac{1}{2}}$
5	1.40	0.198	$\left(\mathrm{erf}\left(100\frac{\langle\eta_{fM}\rangle}{\langle V_p\rangle}\right)\right)^{-\frac{1}{2}}$
6	9.71×10^{-1}	0.170	$\cos\left(\frac{\pi}{200}\langle\kappa\rangle\langle\Omega_\sigma\rangle\right)$
7	3.84×10^{-16}	0.161	$\langle\Omega\rangle\langle V_p\rangle\langle V_p\rangle\frac{\langle\rho_{\mathrm{mol}}\rangle\langle\kappa\rangle}{\left(10^{\sin\left(\frac{\pi}{1000}\frac{\langle r_M\rangle}{R_{\mathrm{Tr}}}\right)\mathrm{erf}\left(\langle G\rangle\langle\alpha\rangle-\frac{1}{20}\right)}\right)^{-\frac{1}{2}}}\times$ $\frac{\langle\theta_{\mathrm{D}}\rangle R_{\mathrm{Tr}}\quad\langle G\rangle\langle\kappa\rangle}{\frac{\langle\alpha\rangle}{\langle\Omega\rangle}\langle\rho_{\mathrm{mol}}\rangle\langle\kappa\rangle\exp\left(\sigma(\chi_{\Delta H})\right)}\times$ $\mathrm{erf}(100r(\alpha))\left(\frac{\langle\rho_{\mathrm{mol}}\rangle}{\langle G\rangle\langle\kappa\rangle\frac{\langle\rho\rangle}{\langle\rho_{\mathrm{mol}}\rangle}\left(\log_{10}\left(\frac{\langle n_{ve}\rangle}{\langle G\rangle}\right)\right)^{-\frac{1}{2}}}\right)^{-\frac{1}{2}}\left(\exp\left(r(\chi_m)\right)\right)^{-\frac{1}{2}}$
8	4.29×10^{-6}	0.121	$\langle B\rangle\langle\theta_D\rangle$
9	2.08×10^{-1}	0.117	$\sin\left(\frac{\pi}{10}\langle\chi_{\Delta H}\rangle\langle r_M\rangle\right)\sigma(\rho)$
10	-3.27×10^{-1}	-0.111	$\left(10^{\left(\mathrm{erf}\left(\langle\nu\rangle R_{\mathrm{Tr(IV)}}-\frac{1}{20}\right)\right)^{-\frac{1}{2}}\left(\mathrm{erf}\left(100\left(\log_{10}\left(\langle\rho\rangle\langle\nu\rangle\right)\right)^{-\frac{1}{2}}\sin\left(\frac{\pi}{1000}\sigma(\theta_{\mathrm{D}})\right)\right)\right)^{-\frac{1}{2}}}\right)^{-\frac{1}{2}}$

11	-2.43×10^{-6}	-0.0978	$\left(\log_{10}\left(\frac{\langle M\rangle}{R_{\mathrm{Tr}}}\right)\right)^{-\frac{1}{2}}\langle n_{ve}\rangle\langle\theta_{\mathrm{D}}\rangle\cos\left(\frac{1}{1000}\langle M\rangle\langle\theta_{\mathrm{D}}\rangle\right)$
12	4.29×10^{-2}	0.0793	$\cos\left(\pi\langle\chi_{\Delta H}\rangle\langle\rho\rangle\right)$
Intercept	-3.34		

Supplementary Table 3 provides a representative symbolic regression model for $log_{10}(w/\langle M\rangle)$. The terms are listed in descending order of the magnitude of their z-scored coefficients, so that the relative importance of each contribution to the model can be directly assessed. This ordering helps visualize which descriptor combinations most strongly govern the predicted hydrogen capacity.

b. Symbolic Model for $\log_{10} P_{eq,RT}$

Supplementary Table 4 An example of symbolic model for $\log_{10} P_{eq,RT}$. Here, $\chi_{\Delta H} = \chi - \chi_H$.

Term index *i*	Coefficient c_i	*Z*-scored coefficient $z(c_i)$	Term t_i
1	5.13×10^{-1}	3.841	$\langle G\rangle R_{\text{Tr + RE}}$
2	1.19×10^{9}	1.929	$\sin\left(\frac{\pi}{20}\frac{\langle Z\rangle}{\langle r_M\rangle}\frac{\langle\alpha\rangle}{\langle\theta_{\text{D}}\rangle}\right)$
3	-8.68×10^{-5}	-1.778	$\frac{\frac{\langle G\rangle\langle\theta_{\text{D}}\rangle}{\langle\nu\rangle}}{R_{\text{Tr + RE}}}$
4	1.76×10^{2}	1.490	$\frac{\langle\rho_{\text{mol}}\rangle}{R_{\text{Tr + RE}}}$
5	7.22×10^{10}	1.328	$\left(\exp\left(\frac{\frac{\langle\alpha\rangle}{\langle\theta_{\text{D}}\rangle}}{\frac{\langle M\rangle}{\langle\rho_{\text{mol}}\rangle}}\right)\right)^{-\frac{1}{2}}$
6	-6.18×10^{1}	-1.001	$\sin\left(\frac{\pi}{2000}\langle B\rangle\right)$
7	-9.58×10^{3}	-0.898	$\langle G\rangle\langle\alpha\rangle$
8	-2.39	-0.779	$\left(\log_{10}\left(\langle M\rangle\langle Z\rangle\right)\right)^{-\frac{1}{2}}$
9	-3.85×10^{-2}	-0.750	$\sigma(\theta_{\text{D}})$
10	1.33×10^{-6}	0.705	$\langle n_{ve}\rangle\langle B\rangle\frac{\langle\kappa\rangle}{\langle\Omega_\sigma\rangle}$
11	9.78×10^{-4}	0.468	$\langle\chi_{\Delta H}\rangle\langle M\rangle\frac{\langle r_M\rangle}{\langle\Omega_\sigma\rangle}$
12	1.57	0.456	$\sin\left(\frac{\pi}{20}\text{erf}\left(\frac{1}{10}r(B)\right)\langle\chi_{\Delta H}\rangle\langle M\rangle\right)$
13	-3.92×10^{-2}	-0.379	$\langle G\rangle R_{\text{Tr(IV)}}$
14	-1.17	-0.288	$\sigma(Z)$
15	9.06×10^{-2}	0.213	$\frac{\langle Z\rangle}{\langle r_M\rangle}r(\chi_{\Delta H})$
16	-1.09×10^{-2}	-0.204	$\langle\chi_{\Delta H}\rangle\langle M\rangle\sigma(\rho)$
17	-4.75×10^{-3}	-0.204	$\langle\kappa\rangle r(n_{ve})$

18	6.72×10^{-2}	0.194	$\left(\sigma(n_c)\right)^{-\frac{1}{2}}$
19	1.18×10^{-1}	0.0956	$r(\kappa)$
Intercept	-7.22×10^{10}		

Supplementary Table 4 provides a representative symbolic regression model for $\log_{10} P_{eq,RT}$. The terms are listed in descending order of the magnitude of their z-scored coefficients, so that the relative importance of each contribution to the model can be directly assessed. This ordering helps visualize which descriptor combinations most strongly govern the predicted hydrogen capacity.

c. Error Distributions of Stacking-ensembled Models for $\log_{10}(w/\langle M\rangle)$ and $\log_{10} P_{eq,RT}$

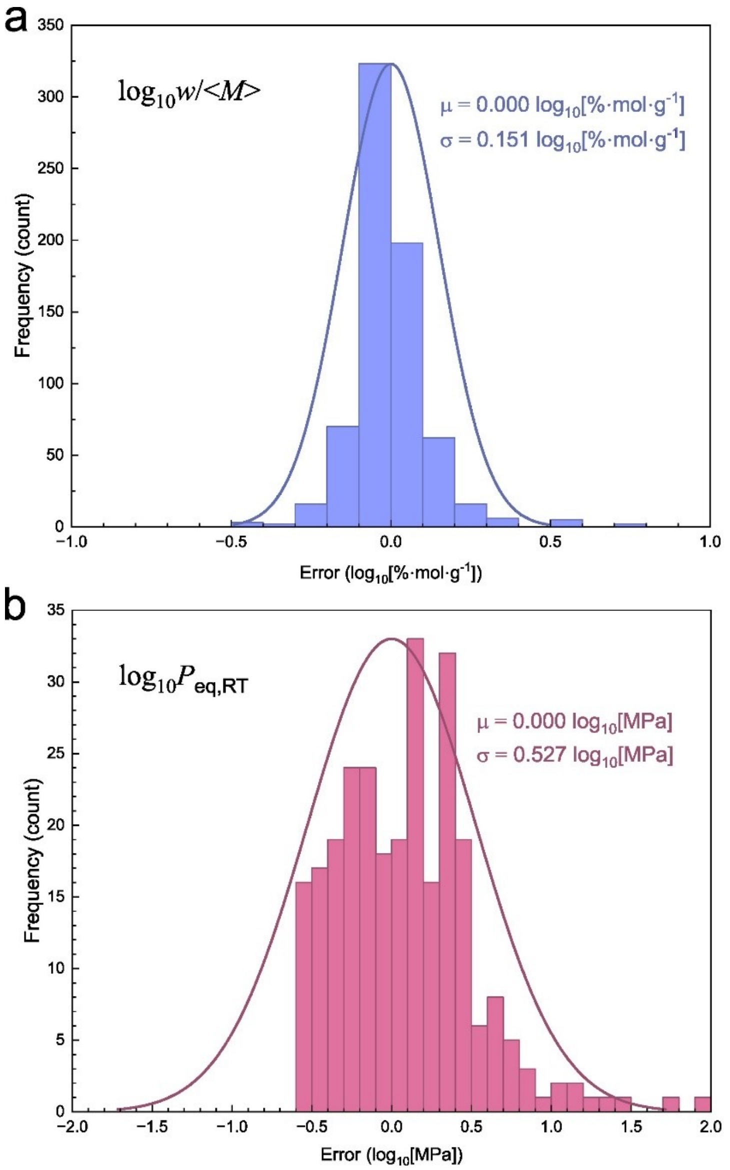

Supplementary Figure 4 Error distributions of stacking-ensembled models for $\log_{10}(w/\langle M\rangle)$ and $\log_{10} P_{eq,RT}$. Error distributions for (a) $\log_{10}(w/\langle M\rangle)$ and (b) $\log_{10}(w/\langle M\rangle)$ obtained from the stacking-ensembled symbolic regression models. Solid curves represent fitted normal distributions, with the mean (μ) and standard deviation (σ) indicated in each panel.

Both error distributions are centered around zero, indicating the absence of systematic bias in the models (see **Supplementary Figure 4**). These results confirm that the models provide reliable and unbiased predictions across the dataset.

d. Stacking-ensembled Symbolic Models for $\log_{10} w$ and $\log_{10}(w\langle M\rangle)$

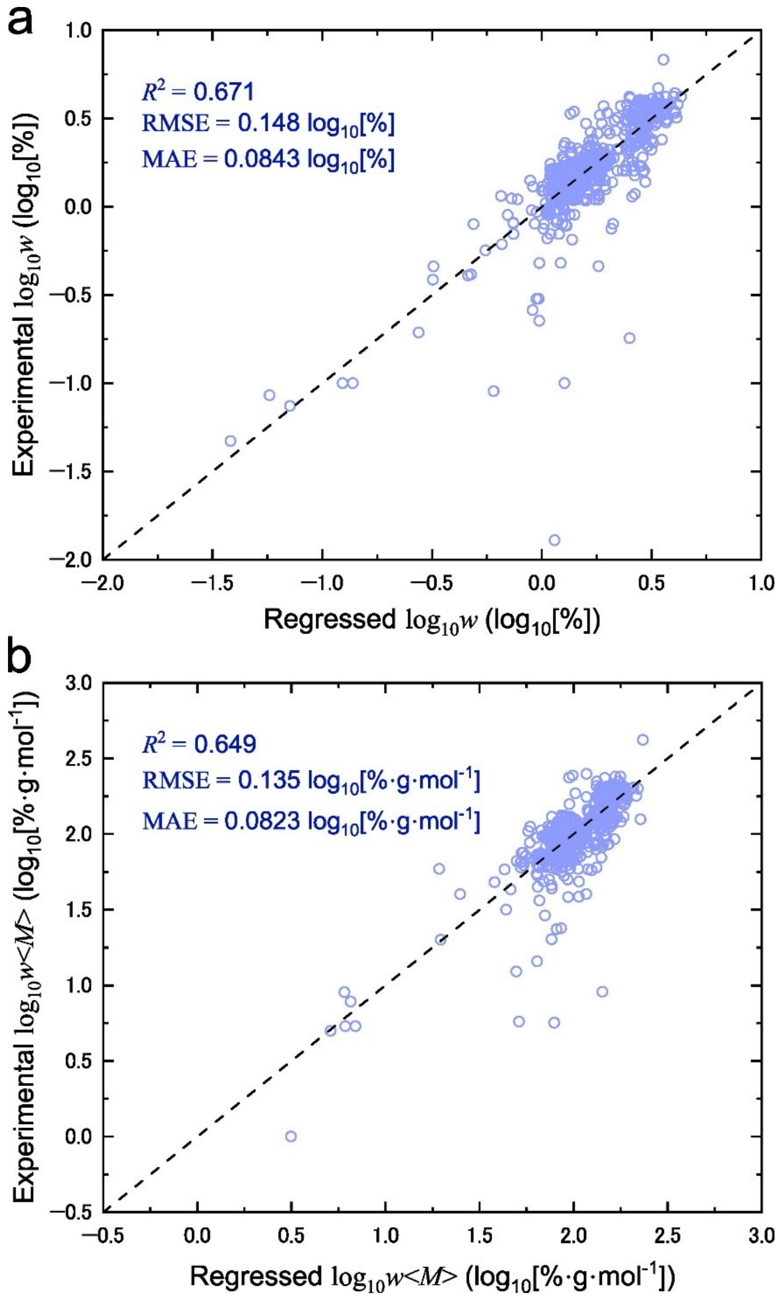

Supplementary Figure 5 Stacking-ensembled symbolic models for $\log_{10} w$ and $\log_{10}(w\langle M\rangle)$. Parity plots for (a) $\log_{10} w$ and (b) $\log_{10}(w\langle M\rangle)$ obtained using stacking-ensembled symbolic regression models. Model performance metrics (R^2, $RMSE$, and MAE) are indicated in each panel.

Compared to the model using $\log_{10}(w/\langle M\rangle)$, both $\log_{10} w$ and $\log_{10}(w\langle M\rangle)$ exhibit lower predictive performance, as reflected by reduced R^2 values and broader scatter in the parity plots (see **Supplementary Figure 5**). This improvement reflects the differing roles of $\langle M\rangle$ across target definitions; whereas $w\langle M\rangle$ penalizes heavy-element systems and $w\langle M\rangle$ favors them, $w/\langle M\rangle$ suppresses mass dependence, thereby enabling clearer extraction of intrinsic structure-property relationships. This difference highlights the strong influence of $\langle M\rangle$ on these target definitions, which can obscure underlying structure-property relationships. As a result, normalization by $\langle M\rangle$ in the form of $w/\langle M\rangle$ provides a more effective representation for data-driven modeling.

Supplementary References

S1. Jang, S.-H. GoodRegressor: A hierarchical inductive bias for navigating high-dimensional compositional space. *arXiv*, February 20, 2026. DOI: 10.48550/arXiv.2510.18325

S2. Hilt, D. E.; Seegrist, D. W. *Ridge, a computer program for calculating ridge regression estimates*; Dept. of Agriculture, Forest Service, Northeastern Forest Experiment Station, 1977.

S3. Zou, H.; Hastie, T.; Tibshirani, R. Sparse principal component analysis. *J. Comput. Graph. Stat.* **2006,** *15* (2), 265-286. DOI: 10.1198/106186006X113430. 265

S4. Cybenko, G. Approximation by superpositions of a sigmoidal function. *Math. Control Signal Systems* **1989,** *2*, 303-314. DOI: 10.1007/BF02551274

S5. Liaw, A.; Wiener, M. Classification and regression by randomForest. *R News* **2002,** *2*, 18-22.

S6. Chen, T.; Guestrin, C. XGBoost: A scalable tree boosting system. *KDD '16: Proceedings of the 22nd ACM SIGKDD International Conference on Knowledge Discovery and Data Mining,* San Francisco, California, USA, August 13-17, 2016; Krishnapuram, B.; Shah, M.; Smola, A.; Aggarwal, C.; Shen, D.; Rastogi, R., Eds.; Association for Computing Machinery: New York, NY, United States, 2017; pp 785-794. DOI: 10.1145/2939672.2939785

S7. Izu, T. *et al*. LightGBM: A highly efficient gradient boosting decision tree. *NIPS'17: Proceedings of the 31st International Conference on Neural Information Processing Systems*, Long Beach, California, USA, December 4-9, 2017; von Luxburg, U.; Guyon, I.; Bengio, S.; Wallach, H.; Fergus, R. Eds.; Curran Associates Inc., Red Hook, New York, United States, 2017; pp 3149-3157. DOI: 10.5555/3294996.3295074

S8. Kim, S.; Lu, P, Y.; Mukherjee, S.; Gilbert, M.; Jing, L.; Čeperić, V. Integration of neural network-based symbolic regression in deep learning for scientific discovery. *IEEE. Trans. Neural. Netw. Learn. Syst.* **2021,** *32* (9), 4166-4177. DOI: 10.1109/TNNLS.2020.3017010

S9. Tenachi, W.; Ibata, R.; Diakogiannis, F. I. Deep symbolic regression for physics guided by units constraints: Toward the automated discovery of physical laws. *ApJ.* **2023,** *959* (2), 99. DOI: 10.3847/1538-4357/ad014c
